# 15-Deoxy-Δ^12,14^-Prostaglandin J_2_ Inhibits Macrophage Colonization by *Salmonella enterica* Serovar Typhimurium

**DOI:** 10.1371/journal.pone.0069759

**Published:** 2013-07-26

**Authors:** Michelle M. C. Buckner, L. Caetano M Antunes, Navkiran Gill, Shannon L. Russell, Stephanie R. Shames, B. Brett Finlay

**Affiliations:** 1 Michael Smith Laboratories, The University of British Columbia, Vancouver, British Columbia, Canada; 2 Department of Microbiology and Immunology, The University of British Columbia, Vancouver, British Columbia, Canada; Universite de la Mediterranee, France

## Abstract

15-deoxy-Δ^12,14^-prostaglandin J_2_ (15d-PGJ_2_) is an anti-inflammatory downstream product of the cyclooxygenase enzymes. It has been implicated to play a protective role in a variety of inflammatory mediated diseases, including rheumatoid arthritis, neural damage, and myocardial infarctions. Here we show that 15d-PGJ_2_ also plays a role in *Salmonella* infection. *Salmonella enterica* Typhimurium is a Gram-negative facultative intracellular pathogen that is able to survive and replicate inside phagocytic immune cells, allowing for bacterial dissemination to systemic sites. *Salmonella* species cause a wide range of morbidity and mortality due to gastroenteritis and typhoid fever. Previously we have shown that in mouse models of typhoid fever, *Salmonella* infection causes a major perturbation in the prostaglandin pathway. Specifically, we saw that 15d-PGJ_2_ production was significantly increased in both liver and feces. In this work we show that 15d-PGJ_2_ production is also significantly increased in macrophages infected with *Salmonella*. Furthermore, we show that the addition of 15d-PGJ_2_ to *Salmonella* infected RAW264.7, J774, and bone marrow derived macrophages is sufficient to significantly reduce bacterial colonization. We also show evidence that 15d-PGJ_2_ is reducing bacterial uptake by macrophages. 15d-PGJ_2_ reduces the inflammatory response of these infected macrophages, as evidenced by a reduction in the production of cytokines and reactive nitrogen species. The inflammatory response of the macrophage is important for full *Salmonella* virulence, as it can give the bacteria cues for virulence. The reduction in bacterial colonization is independent of the expression of *Salmonella* virulence genes SPI1 and SPI2, and is independent of the 15d-PGJ_2_ ligand PPAR-γ. 15d-PGJ_2_ also causes an increase in ERK1/2 phosphorylation in infected macrophages. In conclusion, we show here that 15d-PGJ_2_ mediates the outcome of bacterial infection, a previously unidentified role for this prostaglandin.

## Introduction

**Figure 1:**
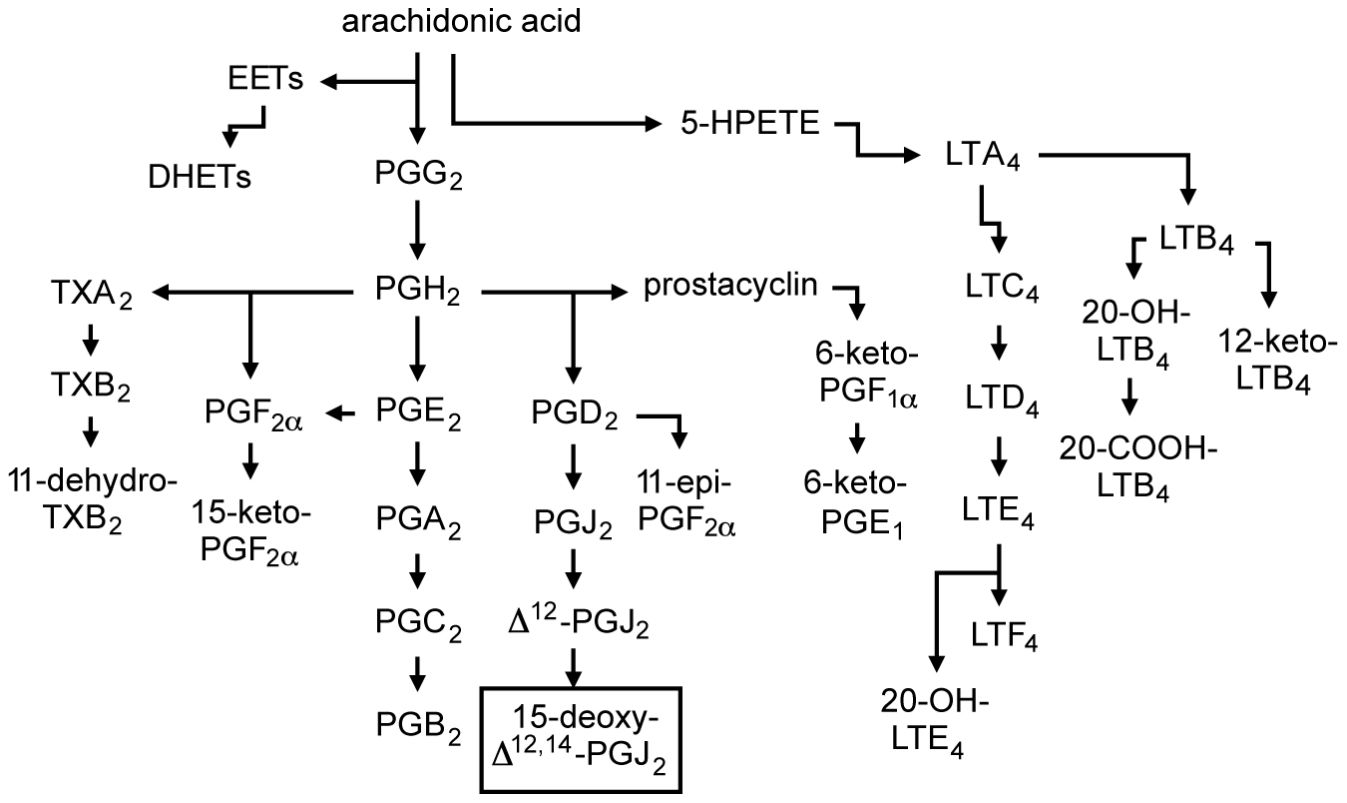
Arachidonic acid metabolism and formation of prostaglandins and leukotrienes. 15d-PGJ_2_ is non-enzymatically produced from PGD_2_.

Prostaglandins (PG) are a class of lipid hormones responsible for a wide range of functions within the body. PGs are synthesized from arachidonic acid that is released from the cell membrane by phospholipase A2 and then modified by the cyclooxygenase enzymes (COX1 and COX2) to enter the PG pathway (Figure 1) [1], [2]. COX1 is constitutively active, whereas COX2 is induced under inflammatory conditions [2]. COX2-derived PGs are involved in a variety of pro- and anti-inflammatory processes [2], [3]. The involvement of COX1 and COX2 in regulating inflammation is evidenced by the increased cardiovascular risk associated with the inhibition of COX2 [4], and the increased susceptibility to colitis in mice lacking these two enzymes [5]. Two waves of COX2 activity have been identified: the first (early) activity is associated with the pro-inflammatory response, whereas the second wave mediates the resolution of inflammation [6], and is associated with high levels of PGD_2_ and 15-deoxy-Δ^12,14^-PGJ_2_ (hereafter referred to as 15d-PGJ_2_) [1], [6].

15d-PGJ_2_ has recently been identified as an anti-inflammatory PG. By forming adducts with various molecules within the cell, 15d-PGJ_2_ is able to modulate a variety of cellular signaling pathways [7]. 15d-PGJ_2_ is an endogenous ligand that activates the nuclear receptor peroxisome proliferator-activated receptor gamma (PPAR-γ) transcription factor, thus inhibiting the NF-κB, STAT, and AP1 signaling pathways, and reducing the production of inflammatory mediators such as iNOS, TNFα, and IL-6 [7]–[9]. 15d-PGJ_2_ has also been found to modify the production of reactive nitrogen species (RNS), the NF-κB pathway, heat shock proteins, JNK signaling, ERK signaling, and cytokine production [6], [9]–[20]. Both RAW264.7 macrophages and HeLa epithelial cells do not produce quantifiable amounts of PPAR-γ [8], [15], [18], which is not necessary for the anti-inflammatory effects of 15d-PGJ_2_ in these cells [18]. In addition, for 15d-PGJ_2_ to activate PPAR-γ, it must be present at relatively high concentrations [21]. Several PPAR-γ independent functions of 15d-PGJ_2_ have recently been described [11], [12], [15]–[20], [22]–[25].

15d-PGJ_2_ inhibits the synthesis of iNOS in activated and peritoneal macrophages, which is at least partially dependent on NF-κB [8], [11], [16]. In RAW 264.7 and J774A.1 macrophages, 15d-PGJ_2_ increases ROS formation, which may inhibit phagocytosis and induce apoptosis at later time points [20], [22]. Furthering its role as an anti-inflammatory mediator, 15d-PGJ_2_ reduces the production of cytokines [10], and reduces the recruitment of bone marrow monocytes during liver inflammation [25]. It was also found that 15d-PGJ_2_ reduces the phagocytic activities of bone marrow macrophages (BMMO) *in vitro* [25]. Recently, the use of nanocapsules loaded with 15d-PGJ_2_ has proved an effective strategy to reduce neutrophil migration, IL-1β, TNF-α, and IL-12p70 production during inflammation [26]. In fact, 15d-PGJ_2_ is so vital to the resolution phase of the inflammatory process, that when it is added back to animals treated with COX2 inhibitors, it is sufficient to restore the normal resolution that occurs after inflammation, which is prevented by COX2 inhibitors [6].

Since 15d-PGJ_2_ has been found to reduce inflammation in such a variety of models, it has been explored as a potential therapeutic in a number of inflammatory diseases. Liu and colleges (2012) concluded that since 15d-PGJ_2_ reduces the general activity of both RAW264.7 and J774A.1 macrophages, it has the potential to be an effective therapeutic for inflammatory diseases [20]. More specifically, the role of 15d-PGJ_2_ and its potential applications in therapy have been explored in rheumatoid arthritis, atherosclerosis, myocardial infarctions, cerebral injury, and gastrointestinal inflammation [8], [10], [27]. 15d-PGJ_2_ has also been found to protect enteric glial cells from oxidative stress, to reduce hepatic inflammation and fibrosis, and to reduce symptoms of COPD in rats [24], [25], [27]. 15d-PGJ_2_ may also be useful in the treatment of cancers, as it has been found to inhibit cell growth and tumorigenicity [28]. In a model of periodontitis, 15d-PGJ_2_ nanocapsules were found to reduce inflammation caused by infection with *Actinobacillus actinomycetemcomitans*, but no effect on bacterial colonization was seen [23]. Despite this wealth of knowledge and exploration into the roles of 15d-PGJ_2_ in inflammatory diseases, little is known about 15d-PGJ_2_ in bacterial infections.

15d-PGJ_2_ has been studied in models of sepsis and septic shock. In models of polymicrobial sepsis, 15d-PGJ_2_ treatment leads to increases in blood pressure, reductions in vascular injury, neutrophil infiltration, cytokine production, renal and liver dysfunction and injury, resulting in increased survival [29], [30]. In rat macrophages treated with heat killed *S. aureus* and *E. coli*, 15d-PGJ_2_ treatment leads to reductions in NO production, TBXB_2_ production, and ERK1/2 and NF-κB activity [31]. In bacterial sepsis, PMN migration is reduced, and this was found to be mediated by PPARγ, and 15d-PGJ_2_ treatment reduced PMN adherence to fibrinogen, another aspect of PMN migration [32]. The role of 15d-PGJ_2_ in microglial inflammatory response to *S. aureus* was examined, and 15d-PGJ_2_ was found to inhibit a variety of cytokines including IL-1β, TNFα, IL-12p40, and MCP1, while in this model the levels of PPARγ were unaffected by either 15d-PGJ_2_ or *S. aureus* treatment [33]. The role of 15d-PGJ_2_ in *H. pylori* infected epithelial cells was also studied, and it was found that 15d-PGJ_2_ treatment reduced JAK/STAT signaling, RANTES production, and NADPH oxidase activity [34]. In this study, the involvement of PPARγ was not determined [34]. Interestingly, 15d-PGJ_2_ treatment of mice one day after infection with the influenza virus was found to significantly reduce morbidity and mortality, in a PPARγ dependent fashion [35]. In this study, 15d-PGJ_2_ reduced the production of chemokines and cytokines, as well as reducing viral titers [35]. They also found that 15d-PGJ_2_ decreased inflammatory infiltrate in the lungs and reduced the production of IL-6, TNFα, CCL2, CCL3, CCL4, and CXCL10, but had no effect on IFNγ production [35]. GW9662, a PPARγ specific inhibitor, was used, and this inhibitor abolished the protection afforded by 15d-PGJ_2_ treatment [35]. These studies show the potential use of 15d-PGJ_2_ in a variety of microbial associated disease conditions, however, it seems that there have not been any studies looking at the role of 15d-PGJ_2_ in *Salmonella* infection.

*Salmonella* is a Gram-negative enteric pathogen that is transmitted by contaminated food or water [36]. Once ingested, the bacteria replicate in the small intestine, and in cases of systemic disease, such as typhoid fever, the bacteria cross the intestinal barrier and are taken up by phagocytes [36], [37]. By means of the *Salmonella* Pathogenicity Island 2 (SPI2) type III-secretion system, *Salmonella* is able to replicate inside macrophages in a special vacuole termed the *Salmonella* containing vacuole [37]–[39]. From inside these macrophages, *Salmonella* is able to disseminate to systemic sites such as the spleen and liver, causing severe disease and bacteremia [36].

We have recently performed a high-throughput metabolomics study to determine the effect of *Salmonella enterica* serovar Typhimurium infection of mice on the chemical composition of multiple body fluids and organs [40]. We found that the PG pathway was greatly perturbed by *Salmonella* infection and that 15d-PGJ_2_ production was greatly increased in infected mice [40]. Therefore, we sought to study the impact of this hormone on the pathogenesis of *Salmonella*. In this study we show that 15d-PGJ_2_ production is increased during *Salmonella* infection of cultured macrophages. Additionally, we examined the roles of individual PGs on bacterial colonization of macrophages, and show that 15d-PGJ_2_ causes a marked decrease in *Salmonella* colonization, despite its well-known role in reducing macrophage activity. We also show that, like many activities of 15d-PGJ_2_, this effect is PPAR-γ independent. Furthermore, we present evidence showing that this reduction in colonization is not due to inhibition of SPI2. Altogether, our data shows a novel role for 15d-PGJ_2_ in infectious disease, and provides further evidence for the importance of inflammation to *Salmonella* pathogenesis.

## Materials and Methods

### Chemical reagents

Streptomycin and dimethyl sulfoxide (DMSO) were purchased from Sigma-Aldrich (St. Louis, USA). 15d-PGJ_2_ was obtained from Cayman Chemical (Ann Arbor, USA).

### Tissue culture

RAW264.7 and J774 macrophages, as well as HeLa epithelial cells, were obtained from the American Type Culture Collection (Manassas, USA). Cells were grown in Dulbecco's Modified Eagle Medium (DMEM; HyClone, Waltham, USA) supplemented with 10% fetal bovine serum (FBS; HyClone), 1% non-essential amino acids (Gibco, Carlsbad, USA) and 1% GlutaMAX (Gibco). Cells were seeded approximately 20 hours before experiments in 24-well plates at a density of 10^5^ cells per well. 15d-PGJ_2_ was dissolved in DMSO and concentrations of 2 µM were used, unless otherwise indicated. Controls without 15d-PGJ_2_ contained the same amounts of DMSO. For infection assays, bacterial cells grown in LB, in mid-logarithmic growth were spun down and resuspended in phosphate-buffered saline (PBS) and diluted in tissue culture medium. Cells were infected at a multiplicity of infection of 10 for 30 minutes at 37°C, 5% CO_2_. Subsequently, cells were washed with PBS and incubated at 37°C, 5% CO_2_ in growth medium containing 100 µg/mL gentamycin (Sigma-Aldrich) for 1 hour. Medium was replaced to decrease the gentamycin concentration to 10 µg/mL for later time points. All media contained (or did not contain for controls) the indicated concentration of 15d-PGJ_2_. At the appropriate times, supernatants were collected and cells were lysed in 250 µL of 1% Triton X-100 (BDH, Yorkshire, UK), 0.1% sodium dodecyl sulfate (Sigma-Aldrich). Serial dilutions were plated on LB plates containing 100 µg/mL of streptomycin (Sigma-Aldrich) for bacterial enumeration. For fold replication assays, CFUs were determined at 2 and 24 hours post-infection and fold replication was calculated by dividing the number of CFUs at 24 hours by the average of the corresponding 2-hour CFU counts.

### Bone marrow macrophage (BMMO) collection and infection

Age-matched C57BL/6 female mice were euthanized by CO_2_ asphyxiation and femurs were removed. Femurs were cleaned, and marrow was removed in Hank's balanced salt solution (Gibco) with 2% FBS. Animal experiments were approved by the Animal Care Committee of the University of British Columbia and performed in accordance with institutional guidelines. Cells were spun down and resuspended in BMMO media [DMEM (HyClone), 20% FBS, 2 mM Glutamax, 1 mM Sodium Pyruvate (Gibco), (5%) penicillin/streptomycin (Gibco), 20% L-conditioned media]. Cells were grown for 7–10 days before use. For infection, BMMO's were seeded in 24-well plates at 1×10^6^ cells/well in BMMO media without penicillin/streptomycin and L-conditioned media. BMMOs were infected with *Salmonella* at a multiplicity of infection of 10, and the gentamycin protection assay was completed as above. CFU was determined at 2, 6, and 10 hours post-infection.

### Cytokine analysis

Cytometric bead assay (CBA) for mouse inflammation (BD Biosciences) was performed following the recommended assay procedure. Supernatants from macrophage infections were used for CBAs.

### Enzyme-linked immunosorbent assays (ELISAs)

ELISAs were performed on culture supernatants from uninfected and infected cells using commercially available ELISA kits to determine concentrations of 15d-PGJ_2_ (Assay Designs, Ann Arbor, USA). ELISAs (BD Biosciences) were also used to examine the concentrations of cytokines (TNF-α, MCP1, IL-10, IL-6) in the supernatants of infected, 15d-PGJ_2_-treated and untreated macrophages. Manufacturer's recommendations and procedures were followed for all ELISAs.

### Quantitative Real-time PCR (qRT-PCR)

RNA was purified using the RNeasy Mini Kit (Qiagen, Hilden, Germany), with the on-column DNA digestion (Qiagen). cDNA was synthesized using the QuantiTect Reverse Transcription Kit (Qiagen). For qRT-PCRs, we used the QuantiTect SYBR Green PCR Kit (Qiagen) and the Applied Biosystems (Foster City, USA) 7500 system. Reactions contained forward and reverse primers at 0.4 µM each. All results were normalized using the mRNA levels of the acidic ribosomal phosphoprotein PO as baseline. Averages of the data obtained with untreated samples were normalized to 1 and the data from each sample (untreated or treated) was normalized accordingly. Primer sequences are available upon request.

### Immunofluorescence microscopy

Macrophages were seeded as mentioned previously, but on glass coverslips. Infections were carried out as above. Cells were fixed using 4% paraformaldehyde (Canemco Supplies, Quebec, Canada) overnight. Cells were then stained using a rabbit, polyclonal, anti-*Salmonella* LPS antibody (BD Biosciences). Prolong Gold containing DAPI (Invitrogen) was used to attach coverslips to the slides. The Zeiss Axioplan Fluorescence Microscope was then used to enumerate the bacteria in each infected macrophage for a total of 50 infected macrophages per sample.

### Trypan blue exclusion

At the appropriate time points after infection, macrophages were released from the bottom of plates using cell scrapers, and stained with Trypan Blue (Gibco). The number of cells were then counted using the Countess automated cell counter (Invitrogen).

### LDH release assay

CytoTox96 Non-Radioactive Cytotoxicity Assay (Promega) was performed on supernatants from infected or uninfected, 15d-PGJ_2_ treated or untreated macrophages. The manufacturer's protocol was followed.

### *Salmonella* growth in 15d-PGJ_2_

*Salmonella* was grown in LB overnight with aeration at 37°C in the presence or absence of 15d-PGJ_2_. *Salmonella* was also grown in DMEM with or without 15d-PGJ2, without aeration, in 5% CO_2_ at 37°C for the indicated time points. Bacterial growth was monitored through measurements of absorbance at 600 nm.

### *hilA, phoP, ssrA* reporter assays

*Salmonella* strains containing fusions between the promoters of *hilA*, *ssrA* or *phoP* and *gfp*, as previously described [41] were sub-cultured in liquid LB culture for 4 hours in the absence or presence of 2 µM 15d-PGJ_2_, and GFP production was analyzed through flow cytometry of bacterial cultures using a FACSCalibur (BD Biosciences, Franklin Lakes, NJ), as indicated. All cultures contained carbenicillin (100 µg/ml) and were incubated at 37°C with shaking (225 rpm). In each experiment, 50,000 events were collected per sample. Also, the *ssrA* reporter plasmid was introduced into *Salmonella* strain MCS004, which constitutively expresses the mKO red/orange protein. This strain was then used to infect RAW264.7 macrophages, as indicated above. Macrophages were lysed and bacteria were washed with PBS containing 2% FBS. GFP and RFP production was analyzed through flow cytometry, performed using an LSR II (BD Biosciences), and data were analyzed with FlowJo 8.7 software (TreeStar, Ashland, OR). In each experiment, 100,000 events were collected per sample.

### Reactive nitrogen and oxygen species production

To determine reactive nitrogen species, the Griess reaction was performed on supernatants taken from macrophages infected as indicated above.

### PPAR-γ inhibitor

RAW264.7 macrophages were seeded as above and GW9662 was used at 4 µM, where indicated.

### Protein extraction and ERK1/2 western blot

RAW264.7 macrophages were seeded as above, overnight without 15d-PGJ_2_. 2 hours before infection cells were treated with indicated concentrations of 15d-PGJ_2_, 10 ng/mL of EGF (Sigma), or 10 mM PD98059 (CalBiochem Billerica USA). Uninfected samples were treated either with DMSO, 15d-PGJ_2_, or EGF. EGF treated samples were used as a positive control for ERK1/2 phosphorylation. PD98059, a MEK inhibitor, was used as a negative control for prevention of ERK1/2 phosphorylation, in the presence of *Salmonella*. Macrophages were infected for 1 hour, then washed with PBS, and lysed in 50 µL of lysis buffer (PBS, 1% Triton X-100 (BDH), 0.1% sodium dodecyl sulfate (Sigma-Aldrich), with protease inhibitor (Roche), and sodium orthovanandate (Sigma). Lysates were collected and spun at 4°C for 20 minutes, supernatants were collected, Bradford assays were performed, and SDS-PAGE loading buffer containing DTT (Sigma) was added. Samples were boiled for 5 minutes, then proteins were separated using denaturing SDS-PAGE. Proteins were then transferred to methanol-activated polyvinylidene difluoride membranes (Bio-Rad, Hercules USA) using wet transfer. Membranes were blocked with rocking for 1 hour using 5% nonfat milk in TBST (Tris-Buffered saline with 0.1% Tween 20). Primary antibodies were added to blocking buffer at 1∶1,000 (Phospho and total p44/42 Map Kinase (ERK1/2) (Cell signaling Techologies, Danvers USA) and anti-Calnexin (Enzo Life Sciences, Farmingdale USA)), and membranes were incubated at 4°C over night with rocking. Membranes were washed 3 times with TBST, then incubated with 1∶5,000 dilution of goat anti-rabbit horseradish peroxidase-conjugated antibodies for 1 hour with rocking in blocking buffer. Membranes were washed 3 times with TBST, then Immun-Star Western C kit (Bio-Rad) was used. Imaging was performed on Bio-Rad ChemiDoc MP Imaging System, and Image Lab (Bio-Rad) software was used.

### Statistical analysis

Data were analyzed by unpaired *t* tests with 95% confidence intervals using GraphPad Prism version 4.0 (GraphPad Software Inc., San Diego, USA).

## Results

### *Salmonella* infection induces 15d-PGJ_2_ production

**Figure 2:**
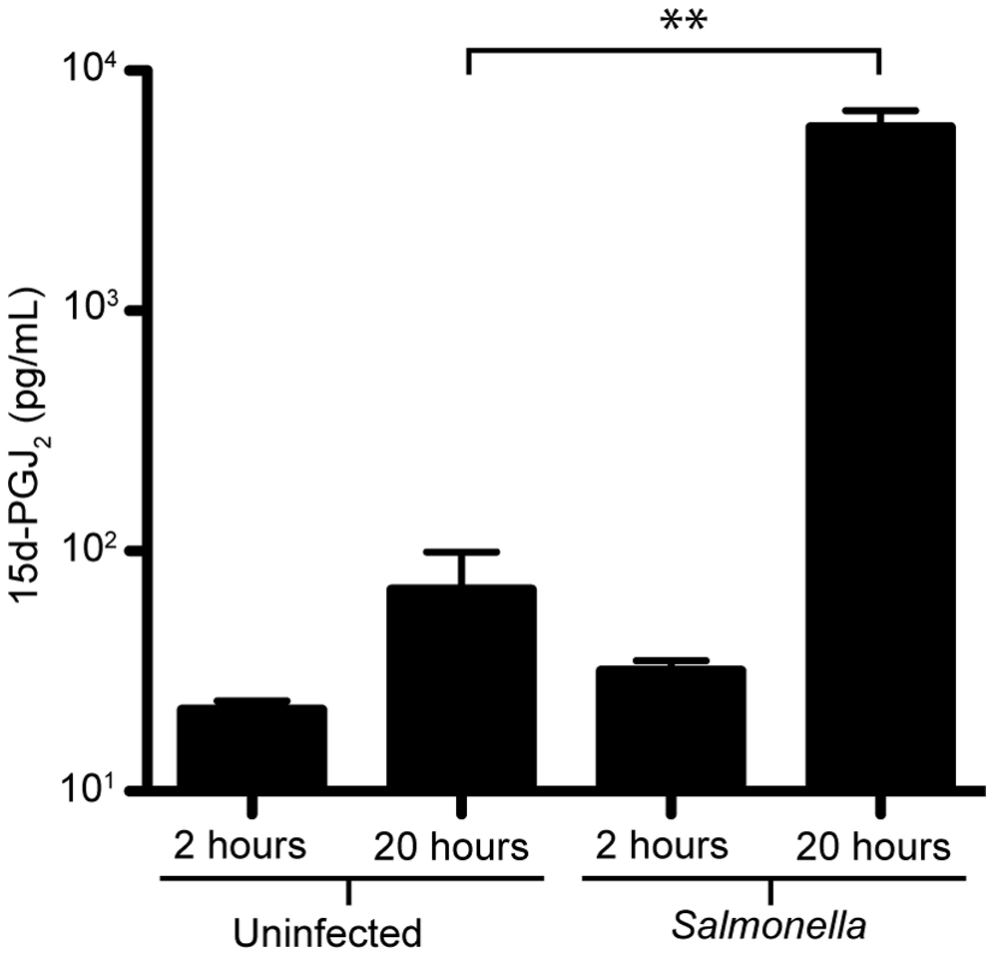
15d-PGJ_2_ is increased during *Salmonella* infection of RAW264.7 macrophages at 20 hours post-infection. Supernatants were collected at 2 and 20 hours post-infection and levels of 15d-PGJ_2_ were determined through ELISA. Results shown are averages of four measurements, with standard errors of means. (**p<0.001).

We have previously shown that the prostaglandin pathway is perturbed in mice infected with *Salmonella* [40]. Specifically, 15d-PGJ_2_ levels were increased during infection in both liver and feces [40]. To further characterize the interactions between the anti-inflammatory molecule 15d-PGJ_2_ and *Salmonella*, so we first established a simplified cell culture system. Because *Salmonella* actively replicates in macrophages, we examined RAW264.7 macrophage cells infected with *Salmonella* to determine if 15d-PGJ_2_ production was induced in these cells, as observed in mice. Similar to mice, we observed a significant increase in the amount of 15d-PGJ_2_ produced by cultured macrophages in response to *Salmonella* (Figure 2).

### Addition of exogenous 15d-PGJ_2_ reduces *Salmonella* colonization of macrophages

**Figure 3:**
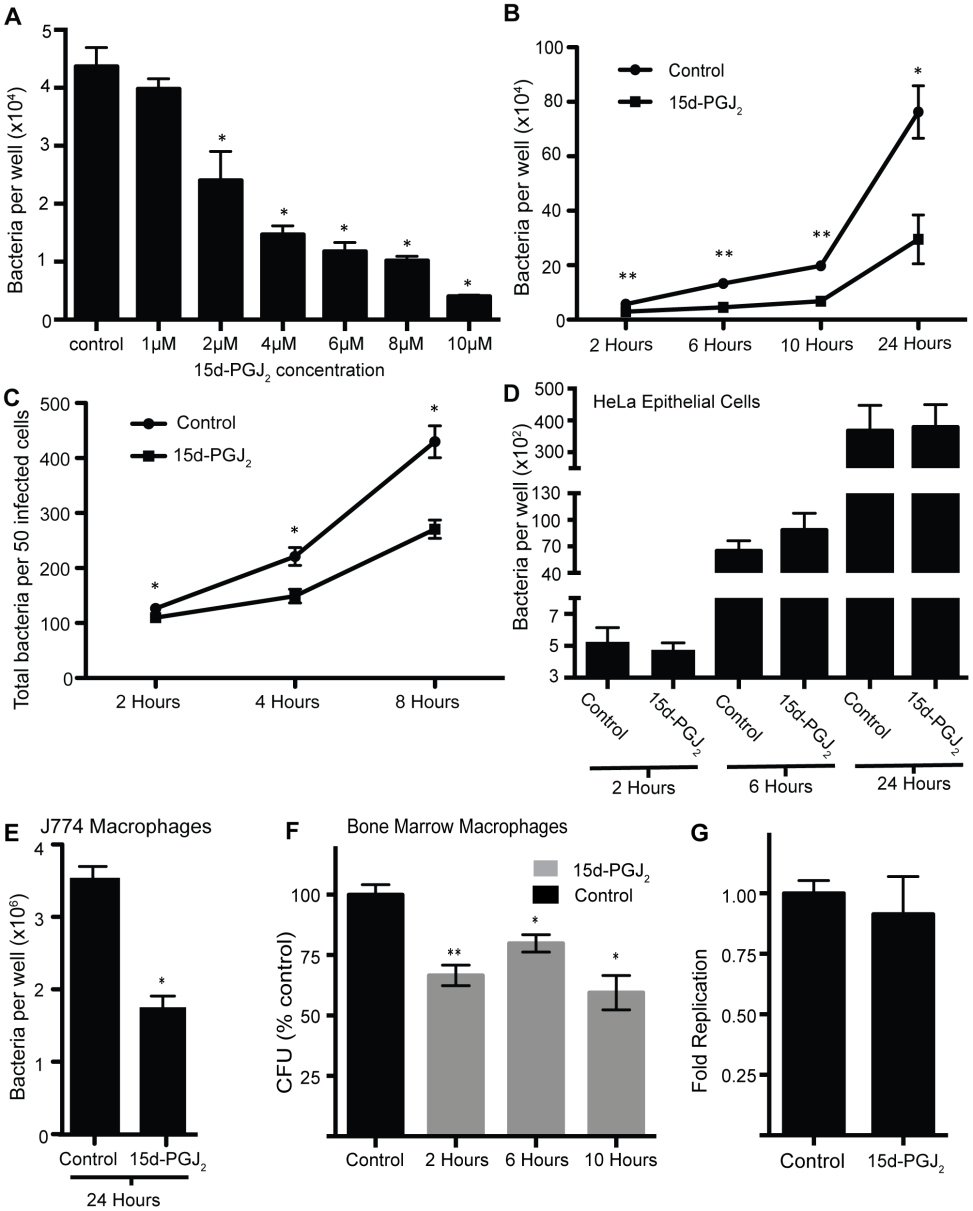
*Salmonella* colonization of macrophages is significantly reduced by the addition of 15d-PGJ_2_. (**A**) *Salmonella* colonization of RAW264.7 macrophages with the addition of increasing concentrations of 15d-PGJ_2_ at 24 hours post infection. (**B**) The effect of 2 µM 15d-PGJ_2_ on *Salmonella* colonization of RAW264.7 macrophages over time as determined by CFU analysis. (**C**) Immunofluorescence microscopy was used to enumerate bacterial colonization in individual macrophages at 2, 4 and 8 hours post-infection. (**D**) *Salmonella* colonization of HeLa epithelial cells treated with 15d-PGJ_2_ at 2, 6, and 24 hours post-infection. (**E**) The effect of 15d-PGJ_2_ on *Salmonella* colonization of J774 macrophages cells, as determined by CFU analysis at 24 hours. (**F**) *Salmonella* colonization of bone marrow macrophages at 2, 6, and 10 hours post-infection, with 15d-PGJ_2_ treatment. (**G**) Fold replication of *Salmonella* in RAW264.7 macrophages treated with 15d-PGJ_2_. Averages of at least 8 measurements are shown with standard errors of means. (*p<0.05, **p<0.001).

Given the role of 15d-PGJ_2_ in reducing the inflammatory response, we wanted to determine if 15d-PGJ_2_ had any effect on *Salmonella* interactions with host cells. To test this, we added increasing concentrations of 15d-PGJ_2_ to RAW264.7 macrophages prior to and during *Salmonella* infection and monitored colonization through bacterial enumeration by selective plating. We saw a dose-dependent decrease in *Salmonella* colonization of macrophages 24 hours post infection (Figure 3a). At the high concentration of 15d-PGJ_2_ cell lifting was slightly increased (data not shown). To determine when during the infection process 15d-PGJ_2_ exerts its effect on *Salmonella* colonization and to understand the kinetics of this phenomenon, we examined bacterial loads in macrophages at 2, 6, 10, and 24 hours post infection in the absence or presence of 2 µM 15d-PGJ_2_. These time points were chosen to give a general overview of *Salmonella* colonization. The time course showed that 15d-PGJ_2_ reduces *Salmonella* colonization as early as 2 hours post infection, and continues to exert its effect until 24 hours post infection (Figure 3b). To confirm that 15d-PGJ_2_ was not killing the macrophages, we used Trypan blue and LDH release assays to measure cell viability. When we used the Trypan blue exclusion assay to count the number of cells in infected, 15d-PGJ_2_ treated and untreated, macrophage cultures, no differences were seen (Figure S1A). An LDH-release assay was also used to ensure that 15d-PGJ_2_ was not causing cell death at 24 hours post-infection. No significant difference was seen in the amount of LDH released by 15d-PGJ_2_ treated cells as compared to untreated control cells (Figure S1B). We also used immunofluorescence microscopy to enumerate the *Salmonella* inside individual macrophages. By counting the bacteria inside 50 macrophages untreated or treated with 2 µM 15d-PGJ_2_ at 2, 4, and 8 hours post infection we saw significantly fewer *Salmonella* in the 15d-PGJ_2_ treated RAW264.7 macrophages (Figure 3c), confirming our CFU observations. Later time points were not used because bacteria became to numerous to accurately count. Therefore, the reduction in *Salmonella* colonization is due to 15d-PGJ_2_ and not to increased macrophage cell death.

### 15d-PGJ_2_ does not inhibit *Salmonella* growth directly

The above results indicate that 15d-PGJ_2_ inhibits *Salmonella* colonization of macrophages. This could occur through a number of distinct mechanisms, the simplest of which would be direct inhibition of bacterial viability and growth. To determine if this was the case, we tested the effect of 15d-PGJ_2_ on *Salmonella* growth in culture media in the absence of macrophages. 15d-PGJ_2_ did not affect the growth of *Salmonella* alone in either LB or DMEM (Figure S2), suggesting that the effect of this hormone on *Salmonella* colonization of macrophages is not due to a direct inhibition of *Salmonella* viability and growth.

### The effect of 15d-PGJ_2_ on *Salmonella* colonization of host cells is dependent on cell type

To determine whether the effect of 15d-PGJ_2_ was dependent on cell type, we infected both HeLa epithelial cells and J774 macrophages with and without 15d-PGJ_2_. We found that 15d-PGJ_2_ had no effect on *Salmonella* colonization of HeLa epithelial cells (Figure 3d), but, like that observed with RAW macrophages, 15d-PGJ_2_ reduced colonization in J774 macrophages (Figure 3e). We also tested the effect of 15d-PGJ_2_ on activated, IFN-γ pre-treated, RAW264.7 macrophages, and found that *Salmonella* colonization was also significantly reduced by 15d-PGJ_2_ treatment (Figure S3). We also wanted to use a model that would more closely represent the murine infection model previously used in our original metabolomics study [40]. To do this, we used bone marrow derived macrophages from C57BL/6 mice, and infected them with *Salmonella* with or without 15d-PGJ_2_ treatment. At 2, 6, and 10 hours post infection there was a significant reduction in *Salmonella* in the 15d-PGJ_2_ treated samples (Figure 3f). Because bone marrow macrophages are highly bactericidal, later time points were not used. Therefore, while 15d-PGJ_2_ significantly reduces *Salmonella* colonization of macrophages, it has no effect on *Salmonella* replication in epithelial cells.

### 15d-PGJ_2_ reduces *Salmonella* entry into macrophages

To determine if 15d-PGJ_2_ was affecting bacterial replication or entry in RAW264.7 macrophages, we determined the fold replication of *Salmonella* (Figure 3G). Fold replication was calculated by comparing CFUs at 2 and 24 hours post-infection. Interestingly, we found that the 15d-PGJ_2_ treated samples had a fold replication similar to control treated samples. This implies that 15d-PGJ_2_ is reducing the entry of *Salmonella* into macrophages.

### 15d-PGJ_2_ affects the immune response of macrophages infected with *Salmonella*

**Figure 4:**
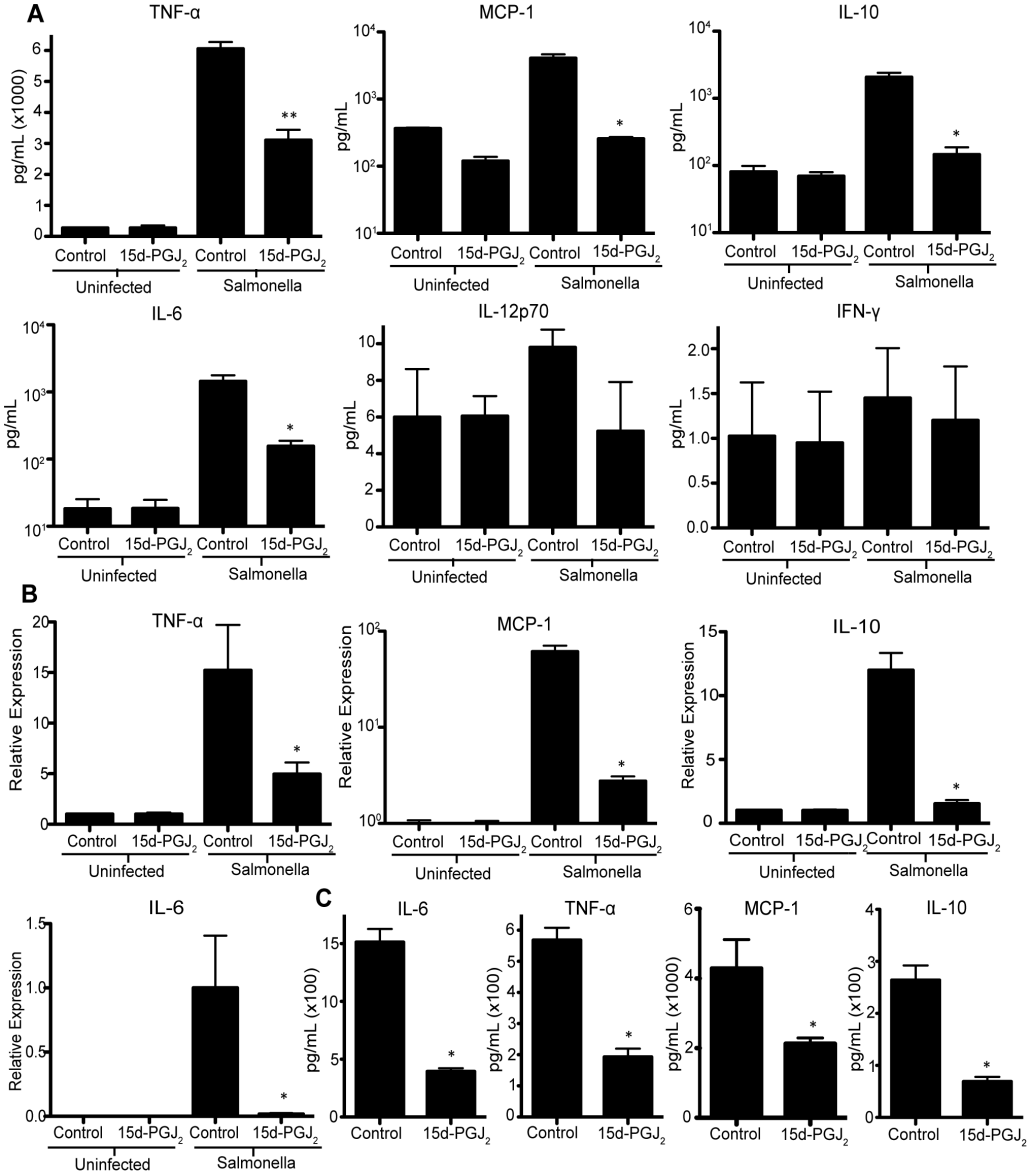
The effect of 2 µM 15d-PGJ_2_ treatment during *Salmonella* infection on cytokine production. RAW264.7 macrophages were examined at 24 hours post infection, cytokine production was determined by; (**A**) CBA assay, (**B**) quantitative real-time PCR, and (**C**) ELISA performed on supernatants from infected cells. Averages of 8 measurements are shown with standard errors of means. (*p<0.05, **p<0.001).

**Figure 5:**
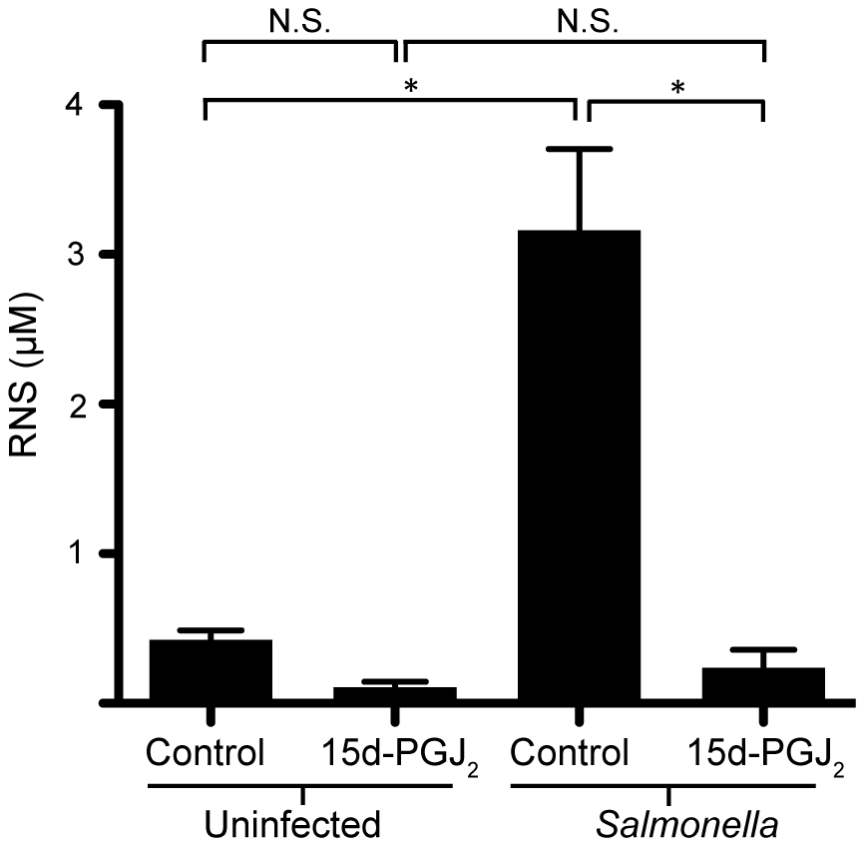
15d-PGJ_2_ reduces the production of reactive nitrogen species. The Griess reaction was used to determine the amount of reactive nitrogen species produced by RAW264.7 macrophages treated with 2 µM 15d-PGJ_2_ and infected with *Salmonella*. Averages of 8 measurements are shown with standard errors of means. (*p<0.05).

As the effect of 15d-PGJ_2_ seemed to be restricted to macrophages, we examined the effects of 15d-PGJ_2_ on the macrophage inflammatory response. By performing a cytometric bead assay (CBA) on supernatants from *Salmonella* infected RAW264.7 macrophages, we found that the 15d-PGJ_2_ treated macrophages produced significantly lower levels of TNF-α, MCP-1, IL-10, and IL-6, whereas levels of IFN-γ and IL-12 were unaffected (Figure 4a). This was confirmed using qRT-PCR (Figure 4b), and ELISA (Figure 4c). IL-12 was also examined using ELISA, and levels were too low to detect (data not shown), corroborating the CBA data. Together, this indicates that 15d-PGJ2 is in fact reducing specific cytokines produced in response to *Salmonella* infection. In addition to reducing the cytokines produced during infection, we also tested whether 15d-PGJ_2_ would reduce other macrophage mechanisms aimed at responding to pathogens. To this end, we show that RNS production in response to *Salmonella* infection was significantly reduced by the addition of 15d-PGJ_2_ (Figure 5).

### 15d-PGJ_2_ does not affect *Salmonella* virulence gene expression

**Figure 6:**
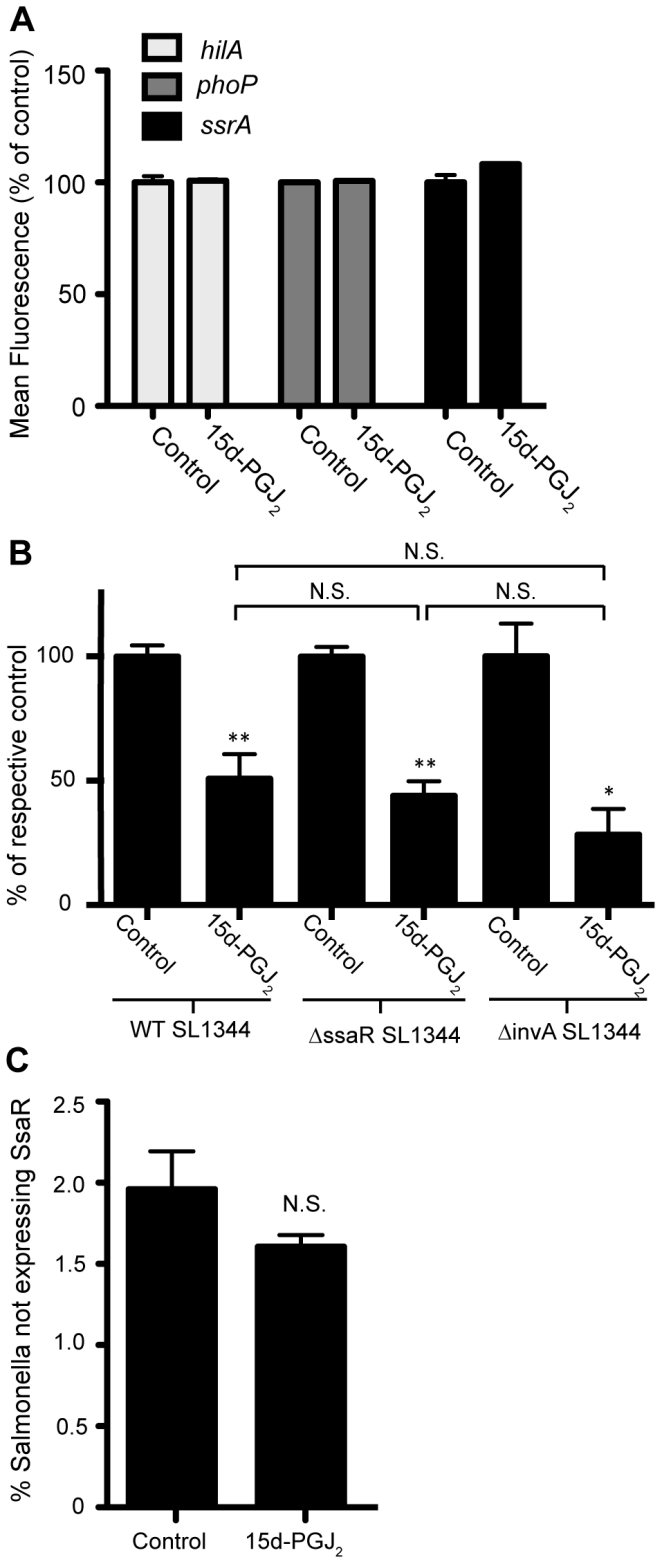
Expression of *Salmonella* virulence genes are unaffected by 15d-PGJ_2_ treatment. (**A**) Wild-type *Salmonella carrying hilA-, phoP-*, and *ssrA-gfp* reporter transcriptional fusions were used to analyze the effect of 15d-PGJ_2_ on virulence gene expression. Cultures were grown in LB for 4 hours. No changes in expression were seen. (**B**) 15d-PGJ_2_ reduced bacterial colonization of macrophages by the wild-type *Salmonella* strain, the Δ*ssaR* strain, and the *ΔinvA* strain. Data are expressed as a percentage of the respective control samples, to illustrate that the extent of the inhibition caused by 15d-PGJ_2_ is equivalent in both strains. (**C**) Flow cytometry analysis of *ssrA* gene expression in *Salmonella* after infection of RAW264.7 macrophages with or without 15d-PGJ_2_ treatment. Averages of 8 measurements are shown with standard errors of means. (*p<0.05, **p<0.001).

We also considered the possibility that in addition to dampening the immune response, 15d-PGJ_2_ may also have an effect on virulence gene expression thus affecting the ability of Salmonella to invade and replicate in macrophages. Therefore, we used *Salmonella* reporter strains to determine if the regulation of virulence genes was directly affected by 15d-PGJ_2_ treatment. For these experiments, we chose the SPI1 regulatory gene *hilA*, the SPI2 regulatory gene *ssrA*, and the two-component regulatory gene *phoP* to examine the expression of virulence genes in the presence of 15d-PGJ_2_, as these genes play major roles in the regulation of the SPI1 and SPI2 virulence regulons during the infection process. To study their expression, reporter fusions between the promoters of these genes and *gfp* were used as previously described [41]. We found that their expression was not affected by the addition of 15d-PGJ_2_ (Figure 6a). Additionally, because SPI2 is highly induced inside the *Salmonella* containing vacuole, where it is known to play a major role in systemic virulence and the formation of a hospitable intracellular niche in phagocytes [36], [39], we determined the activity of SPI2 in 15d-PGJ_2_ treated macrophages. First we infected 15d-PGJ_2_ treated or untreated macrophages with either the wild-type *Salmonella* strain, or the *ΔssaR* strain, which does not secrete any SPI2 effectors into the macrophage. Since we thought that 15d-PGJ_2_ may be affecting *Salmonella* colonization by inhibiting SPI2, we anticipated that infecting host cells with a strain already missing a SPI2 component would abolish the colonization defect seen with 15d-PGJ_2_ treatment. Interestingly, this was not the case; in fact, macrophage colonization by the Δ*ssaR* strain was inhibited to the same extent as the wild-type infections when compared to the samples that did not receive 15d-PGJ_2_ (Figure 6b). We also wanted to test if the pathway by which Salmonella was taken up by the macrophages was being affected by 15d-PGJ_2_ treatment. To this end, we infected macrophages with a *ΔinvA* strain, which does not secrete SPI1 effectors, and therefore bacterial uptake occurs through phagocytosis alone. Our data show that the *ΔinvA* strain's colonization was inhibited by 15d-PGJ_2_ to the same extent as wild-type *Salmonella*. In Figure 6b the data are expressed as a percentage of the respective control samples, to illustrate that the extent of the inhibition caused by 15d-PGJ_2_ is equivalent, even though the Δ*ssaR* and *ΔinvA* strain colonized at a lower levels than the wild-type *Salmonella*. To further ensure that SPI2 expression was not affected in 15d-PGJ_2_ treated macrophages we used the *ssrA* reporter fusion in constitutive mKO expressing bacteria (red/orange), and looked at *ssrA* expression after macrophage infection. Our data did not show any differences in *ssrA* expression in untreated or 15d-PGJ_2_ treated macrophages (Figure 6c). Therefore our data indicates that despite 15d-PGJ_2_ generally reducing the inflammatory response, the expression of virulence genes is not directly affected.

### 15d-PGJ2 affects Salmonella colonization via a PPAR-γ independent mechanism

**Figure 7:**
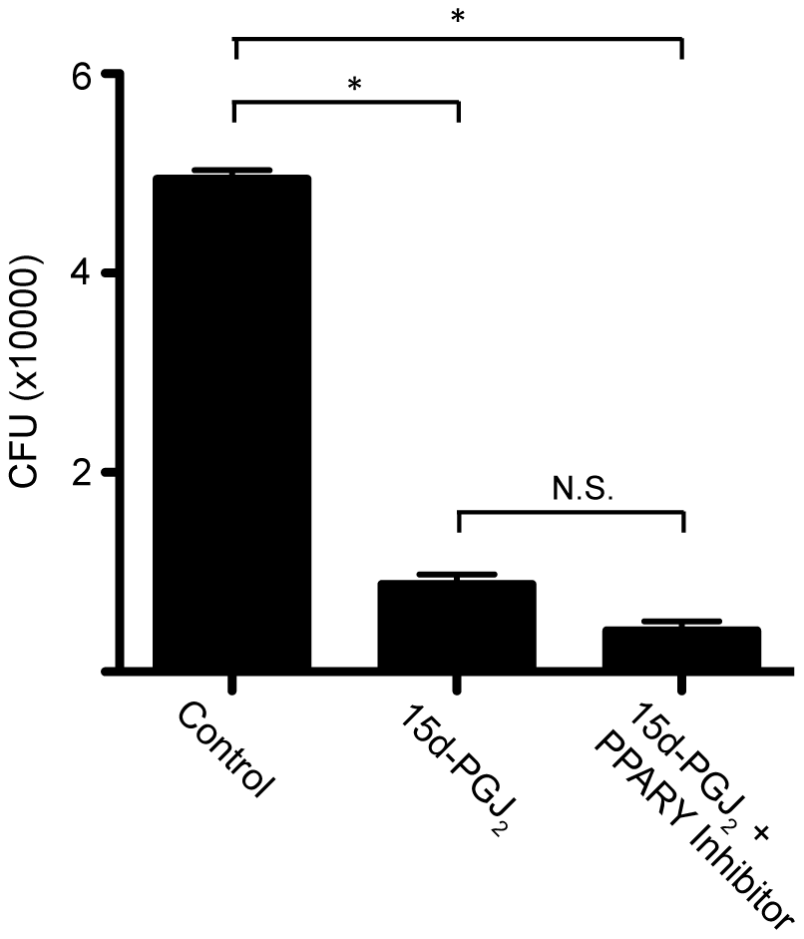
PPAR-γ inhibitor has no effect on *Salmonella* colonization. The effect of the addition of a PPAR-γ inhibitor to 15d-PGJ_2_ treated macrophages infected with *Salmonella* on bacterial colonization. Averages of 8 measurements are shown with standard errors of means. (*p<0.05, **p<0.001).

15d-PGJ_2_ is known to bind to and alter PPAR-γ activity, however, it is also not considered to be important in RAW264.7 macrophages. Ricote *et. al.* have shown that PPAR-γ is not expressed to a significant extent in these cells [8]. We wanted to ensure that PPAR-γ was not involved in our system. To do so, we added the PPAR-γ inhibitor GW9662 to RAW264.7 macrophages infected with *Salmonella* and treated with 15d-PGJ_2_ and monitored bacterial colonization, as above. The inhibitor was unable to restore the colonization defect seen with 15d-PGJ_2_ (Figure 7), indicating that the effect of 15d-PGJ_2_ on macrophage colonization by *Salmonella* may be through a PPAR-γ independent mechanism.

### 15d-PGJ_2_ induces ERK1/2 phosphorylation in macrophages infected with *Salmonella*

**Figure 8:**
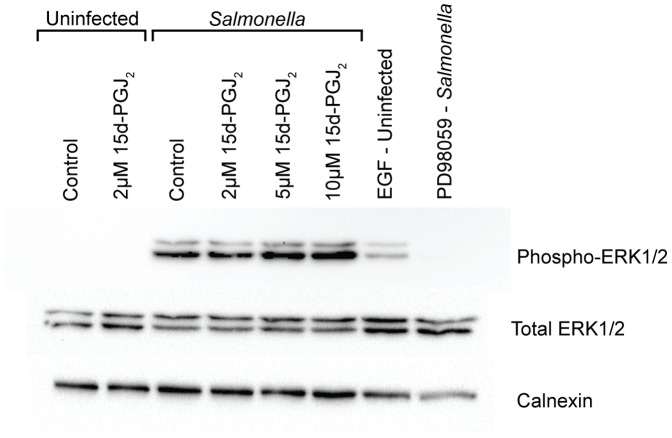
Phosphorylated ERK1/2 levels increase with 15d-PGJ_2_ treatment. Phosphorylated ERK1/2 levels are increased 1 hour after *Salmonella* infection of RAW264.7 macrophages treated with increasing concentrations of 15d-PGJ_2_. Figure is representative of 3 independent experiments.

15d-PGJ_2_ has been shown to alter the activity of ERK1/2. Therefore we examined the phosphorylation of ERK1/2 in RAW264.7 macrophages treated with 15d-PGJ_2_ and infected with *Salmonella* (Figure 8). ERK1/2 phosphorylation was not seen in uninfected samples. ERK1/2 phosphorylation increased with increasing concentrations of 15d-PGJ_2_. EGF was used as a positive control of ERK1/2 phosphorylation, and PD98059, an ERK1/2 inhibitor, was used as a negative control. This indicates that 15d-PGJ_2_ is inducing the activity of ERK1/2. Because increased concentrations of 15d-PGJ_2_ can lead to increased cell death, cells were treated for 2 hours prior to infection, instead of overnight, and no increase was seen in cell lifting (data not shown).

## Discussion

The potential role of 15d-PGJ_2_ during bacterial infection was initially considered because of the results of a metabolomics screen recently performed by our lab [40]. Because we identified the PG pathway to be highly responsive to *Salmonella* infection in our metabolomics analysis, we went on to examine if this pathway played a role in the establishment of infection by *Salmonella*. Here, we show that at 20 hours post-infection macrophages produce high levels of 15d-PGJ_2_ in response to *Salmonella* infection, which coincides with our previous data showing that 15d-PGJ_2_ is highly induced by *Salmonella* infection in mice. We then hypothesized that the high level of 15d-PGJ_2_ production observed would likely have a significant effect during the course of infection. To test this, we added 15d-PGJ_2_ exogenously and monitored its effects on *Salmonella* colonization of macrophages. Our data demonstrated a significant impact of 15d-PGJ_2_ on *Salmonella* burden and also showed a dose-dependent decrease in *Salmonella* colonization, clearly indicating that 15d-PGJ_2_ is sufficient to prevent bacterial colonization of macrophages. Some reports have claimed that 15d-PGJ_2_ treatment causes apoptosis in macrophages [17], and in fact at high concentrations of 15d-PGJ_2_ we did begin to see increased macrophage cell death (data not shown). Therefore, we used 2 µM 15d-PGJ_2_ in our experiments because this was the lowest concentration at which we still saw a decrease in colonization without an increase in cell death (Figure S1).

The effect of 15d-PGJ_2_ on *Salmonella* colonization was not limited to RAW264.7 macrophages. In fact, 15d-PGJ_2_ reduced bacterial colonization in both J774 macrophages and BMMOs. BMMOs are considered more ‘physiologically relevant’, and are able to clear *Salmonella* more rapidly and effectively than RAW264.7 macrophages (data not shown). It is also interesting that 15d-PGJ2 appears to be impacting bacterial entry, as indicated by Figure 3g. Furthermore, 15d-PGJ_2_ did not affect *Salmonella* replication in HeLa epithelial cells. This could indicate that the 15d-PGJ_2_–induced resistance to *Salmonella* is cell type specific. It is possible that 15d-PGJ_2_ alters a macrophage specific response to bacteria, thus inhibiting bacterial infection. It is interesting to note that Straus and colleagues [12] found that 15d-PGJ_2_ had a dramatically different effect on NF-κB inhibition in RAW264.7 and HeLa cells. The *Salmonella* life cycle inside of these two cell types is also very different [36] and this may be the reason for the significantly different responses.

Previously published results indicate that 15d-PGJ_2_ is able to reduce the production of cytokines in response to LPS [20], [27]. Here, we show the same effect with live, replicating bacteria. Specifically, we saw a reduction in IL-10 production in 15d-PGJ_2_ treated cells. IL-10 is increased via a SPI2 dependent mechanism during *Salmonella* infection, and may inhibit ROS and RNS in macrophages [42], [43]. We also saw a reduction in the amount of IL-6 and MCP-1 produced by macrophages treated with 15d-PGJ_2_ and infected with *Salmonella*. Interestingly, we did not see a change in IL-12, which can stimulate IFN-γ production [43]. IFN-γ is very important for the defense against *Salmonella*, and is produced predominantly by NK cells and T cells [43]. Since IFN-γ plays such an important role in anti-*Salmonella* defenses we were surprised to see that 15d-PGJ_2_ did not significantly alter either IL-12 or IFN-γ production. We also show that TNF-α, which is known to be important for anti-*Salmonella* defenses and is involved in triggering NO production [43], [44], was decreased with 15d-PGJ_2_ treatment. Generally these data indicate a reduction in pro-inflammatory molecules.

Similar to the results presented here, Cloutier *et al.* found that 15d-PGJ_2_ treatment reduced the production of IL-6, and TNFα in mice infected with the influenza virus, but also showed no effect on IFN-γ production [35]. In addition, Kielian *et al.*, showed that 15d-PGJ_2_ selectively inhibited the inflammatory response of microglia in response to *S. aureus* [33]. This group showed 15d-PGJ_2_ dependent reduction in the production of IL-12p40, MCP1, and TNFα [33].

There is increasing evidence that *Salmonella* induced inflammation can actually benefit the pathogen in both intestinal colonization and systemic disease. Stecher *et al*. showed that intestinal inflammation is both necessary and sufficient in allowing *Salmonella* to outcompete the microbiota [45]. More specifically, Winter and colleagues (2010) showed that *Salmonella* induced gut inflammation resulted in the production of tetrathionate, which *Salmonella* is able to use as an electron acceptor, thus showing a mechanism by which inflammation benefits this pathogen [46]. *Salmonella* also gains a growth advantage by the production of ethanolamine and nitrate, which *Salmonella* is able to respire [47], [48]. It was also recently shown that *Salmonella* induces the recruitment of neutrophils to the intestinal lumen [49]. These neutrophils produce neutrophil elastase, which shifts the microbiota to favour *Salmonella* colonization [49]. At the systemic level, Arpaia and colleagues (2011) showed that TLR induced innate immunity in response to *Salmonella* induces virulence in the pathogen, allowing bacterial growth leading to systemic disease [50]. These studies, like the one we present here, show that inflammation is an important aspect of *Salmonella* infection.

Another immune mechanism generally considered to be critical to the host's defense against *Salmonella* are RNS. RNS are normally produced during *Salmonella* infection and are integral to bacterial killing as they modify components of the bacterial electron transport chain, metabolic enzymes, transcription factors, DNA, and DNA associated proteins [51]–[53]. Furthermore, IFN-γ pretreated macrophages have a stronger RNS response to *Salmonella* than untreated macrophages [53]. Intriguingly, we see both a reduction in RNS as well as a reduction in *Salmonella* burden in 15d-PGJ_2_ treated macrophages. In addition, we see this effect in both untreated and IFN-γ treated macrophages, which is interesting since IFN-γ treated macrophages are thought to have a much stronger RNS response to *Salmonella*. The reduction in RNS is also in line with the reduction in TNF-α that is caused by 15d-PGJ_2_ addition.

Our data also indicates that the 15d-PGJ_2_ mediated changes in bacterial colonization are SPI2 independent. We initially explored the possibility of SPI2 involvement due to the apparent reduction in the inflammatory response of the macrophages, and were surprised to see that SPI2 does not appear to be involved. We have also examined the potential role of PPAR-γ in the 15d-PGJ_2_ mediated reduction in *Salmonella* colonization. Not surprisingly, we found that the effects of 15d-PGJ_2_ on bacterial colonization were PPAR-γ independent. Furthermore, RAW264.7 macrophages do not appear to produce physiologically relevant amounts of PPAR-γ [8]. However, in the future siRNA knock-down and over-expression strains could be used to ensure that PPAR-γ is not involved in the 15d-PGJ_2_ mediated reduction in *Salmonella* growth.

We have also shown that the levels of phosphorylated ERK1/2 increase with increasing concentrations of 15d-PGJ_2_ treatment of *Salmonella* infected macrophages. *Salmonella* infection is known to lead to the activation of the ERK MAPK pathway [54]. Furthermore, the MEK/ERK pathway regulates changes in the actin cytoskeleton [55]. It is possible that 15d-PGJ_2_ is resulting in dis-regulation of ERK1/2 activity, which reduces *Salmonella* entry into macrophages; however this remains to be elucidated.

Research into the use of 15d-PGJ_2_ for the treatment of inflammatory diseases is already underway, and recently the use of nanocapsules as a mechanism of delivery has shown promise [23], [26]. In the future, the use of these or other delivery mechanisms may provide a way to effectively administer 15d-PGJ_2_ during *Salmonella* infection. Such research may provide insights into a novel mechanism of treating salmonellosis and possibly other bacterial infections. Our work sheds light onto a new role of 15d-PGJ_2_, namely the control of *Salmonella* pathogenesis and replication within phagocytic immune cells. The role of 15d-PGJ_2_ in bacterial infections is uncharacterized, and our work lays the foundation for further research into this area.

## Supporting Information

Figure S1 **Enumeration of live RAW264.7 macrophages (A) using Trypan Blue exclusion after treatment with 2 µM 15d-PGJ_2_ and infection with** ***Salmonella*** **.** (**B**) LDH released from macrophages infected with *Salmonella* in the absence or presence of 15d-PGJ_2_.
(TIF)

Figure S2 ***Salmonella*** **growth curves in (A) LB and (B) DMEM, with and without 2 µM 15d-PGJ_2_ treatment.**
(TIF)

Figure S3 **The effect of 15d-PGJ_2_ on** ***Salmonella*** **colonization of 2 ng/mL IFN-γ activated RAW264.7 macrophages 24 hours post infection.** Averages of 8 measurements are shown with standard errors of means. (*p<0.05).
(TIF)
