# Supplementary figures and images for: 15-Deoxy-Δ12,14-Prostaglandin J2 Inhibits Macrophage Colonization by Salmonella enterica Serovar Typhimurium
Source: PLoS One. 2013 Jul 26;8(7):e69759. doi: 10.1371/journal.pone.0069759 (PMC3724865; doi:10.1371/journal.pone.0069759)

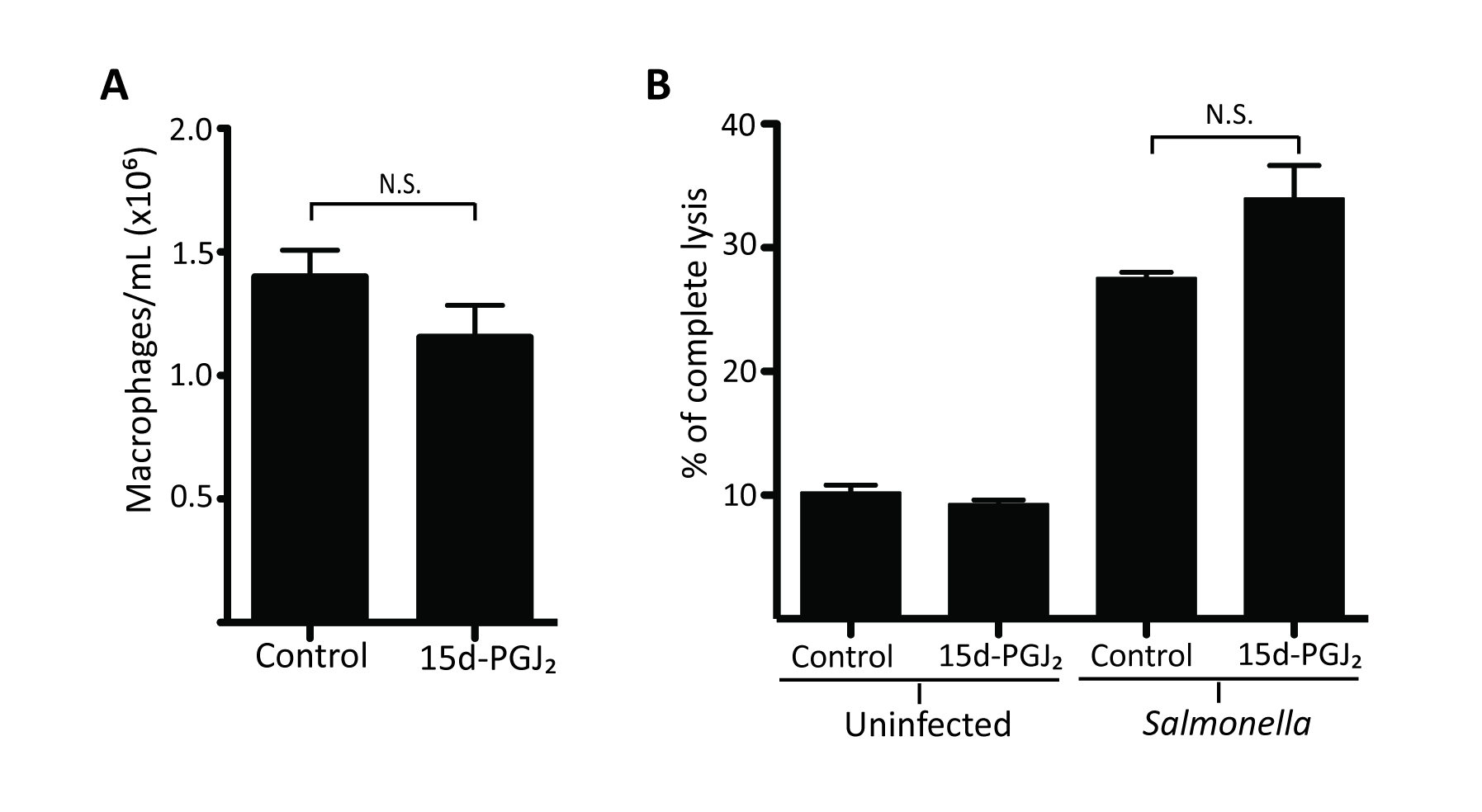

Supplement: Figure S1 — Enumeration of live RAW264.7 macrophages (A) using Trypan Blue exclusion after treatment with 2 µM 15d-PGJ2 and infection with Salmonella . (B) LDH released from macrophages infected with Salmonella in the absence or presence of 15d-PGJ2. (TIF) [file pone.0069759.s001.tif]

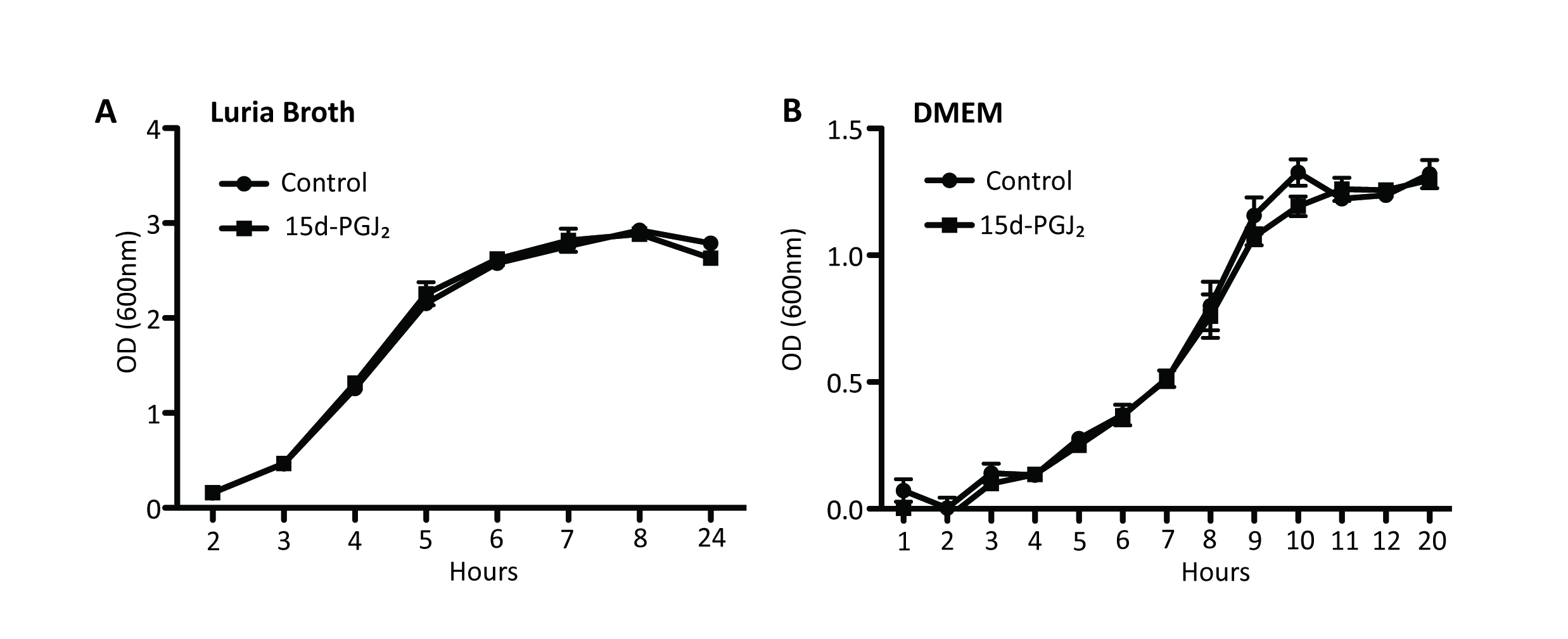

Supplement: Figure S2 — Salmonella growth curves in (A) LB and (B) DMEM, with and without 2 µM 15d-PGJ2 treatment. (TIF) [file pone.0069759.s002.tif]

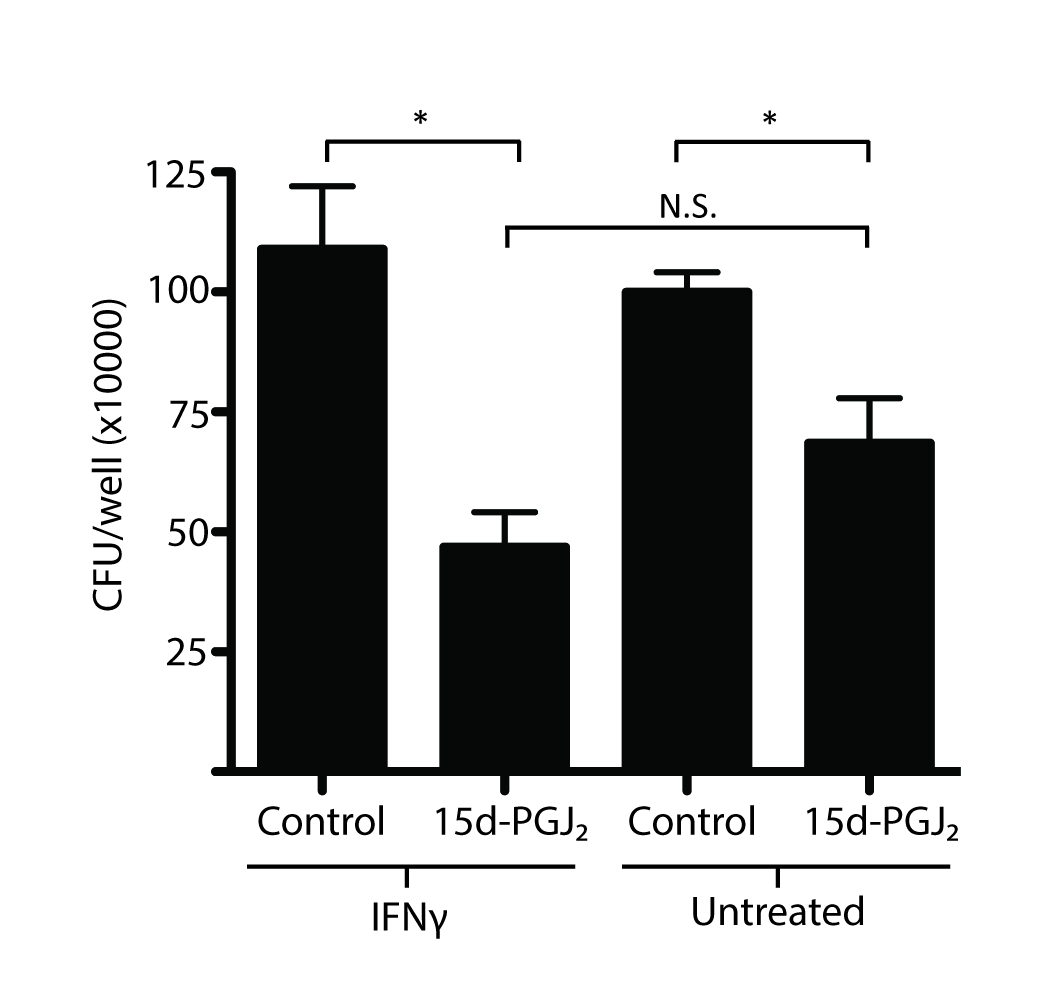

Supplement: Figure S3 — The effect of 15d-PGJ2 on Salmonella colonization of 2 ng/mL IFN-γ activated RAW264.7 macrophages 24 hours post infection. Averages of 8 measurements are shown with standard errors of means. (*p<0.05). (TIF) [file pone.0069759.s003.tif]
